# Biomarkers of Neurological Outcome After Aneurysmal Subarachnoid Hemorrhage as Early Predictors at Discharge from an Intensive Care Unit

**DOI:** 10.1007/s12028-020-01110-2

**Published:** 2020-09-25

**Authors:** Jaroslaw Kedziora, Malgorzata Burzynska, Waldemar Gozdzik, Andrzej Kübler, Katarzyna Kobylinska, Barbara Adamik

**Affiliations:** 1 Department of Anaesthesiology and Intensive Therapy, Wroclaw Medical University, Borowska St. 213, 50-556 Wrocław, Poland; 2 Faculty of Mathematics, Informatics and Mechanics, University of Warsaw, Banacha 2, 02-097 Warsaw, Poland

**Keywords:** Brain-specific biomarkers, Glial fibrillary acidic protein, Microtubule-associated protein tau, Neuron-specific enolase, S100B protein, Subarachnoid hemorrhage

## Abstract

**Background:**

Subarachnoid bleeding is associated with brain injuries and ranges from almost negligible to acute and life threatening. The main objectives were to study changes in brain-specific biomarker levels in patients after an aneurysmal subarachnoid hemorrhage (aSAH) in relation to early clinical findings, severity scores, and intensive care unit (ICU) outcome. Analysis was done to identify specific biomarkers as predictors of a bad outcome in the acute treatment phase.

**Methods:**

Analysis was performed for the proteins of neurofilament, neuron-specific enolase (NSE), microtubule-associated protein tau (MAPT), and for the proteins of glial cells, S100B, and glial fibrillary acidic protein (GFAP). Outcomes were assessed at discharge from the ICU and analyzed based on the grade in the Glasgow Outcome Scale (GOS). Patients were classified into two groups: with a good outcome (Group 1: GOS IV–V, *n *= 24) and with a bad outcome (Group 2: GOS I–III, *n *= 31). Blood samples were taken upon admission to the ICU and afterward daily for up to 6 days.

**Results:**

In Group 1, the level of S100B (1.0, 0.9, 0.7, 2.0, 1.0, 0.3 ng/mL) and NSE (1.5, 2.0, 1.6, 1.2, 16.6, 2.2 ng/mL) was significantly lower than in Group 2 (S100B: 4.7, 4.8, 4.4, 4.5, 6.6, 6.8 ng/mL; NSE: 4.0, 4.1, 4.3, 3.8, 4.4, 2.5 1.1 ng/mL) on day 1–6, respectively. MAPT was significantly lower only on the first and second day (83.2 ± 25.1, 132.7 ± 88.1 pg/mL in Group 1 vs. 625.0 ± 250.7, 616.4 ± 391.6 pg/mL in Group 2). GFAP was elevated in both groups from day 1 to 6. In the ROC analysis, S100B showed the highest ability to predict bad ICU outcome of the four biomarkers measured on admission [area under the curve (AUC) 0.81; 95% CI 0.67–0.94, *p *< 0.001]. NSE and MAPT also had significant predictive value (AUC 0.71; 95% CI 0.54–0.87, *p *= 0.01; AUC 0.74; 95% CI 0.55–0.92, *p *= 0.01, respectively). A strong negative correlation between the GOS and S100B and the GOS and NSE was recorded on days 1–5, and between the GOS and MAPT on day 1.

**Conclusion:**

Our findings provide evidence that brain biomarkers such as S100B, NSE, GFAP, and MAPT increase significantly in patients following aSAH. There is a direct relationship between the neurological outcome in the acute treatment phase and the levels of S100B, NSE, and MAPT. The detection of brain-specific biomarkers in conjunction with clinical data may constitute a valuable diagnostic and prognostic tool in the early phase of aSAH treatment.

## Introduction

Subarachnoid bleeding is associated with brain injuries and ranges from almost negligible to acute and life threatening. An acute brain injury after an aneurysmal subarachnoid hemorrhage (aSAH) leads to the destruction of brain cells and the release of brain-specific proteins to the cerebrospinal fluid and systemic blood circulation. Brain proteins can pass directly through a damaged blood–brain barrier into the systemic circulation, where they can be detected [1, 2]. Therefore, the proteins abundant in the neurons and glia of the central nervous system (CNS) can be sensitive markers of brain damage caused by aSAH in the acute and long-term treatment phases. The severity of the initial brain damage is one of the most important factors associated with outcome after aSAH. Brain ischemia associated with an increase in intracranial pressure (ICP) and focal brain ischemia, caused by tissue compression, are the main mechanisms of initial brain damage after aSAH [3, 4]. Therefore, the identification of biomarkers that are known to be released during brain ischemia after aSAH could be useful in the clinical setting [5]. The concept of brain-specific biomarkers refers to substances found in high concentration in the central nervous system (CNS) and absent or present in low concentration in blood. Proteins abundant in the neurons and glia of the CNS are neuron-specific enolase (NSE), S100B protein, microtubule-associated protein tau (MAPT), and glial fibrillary acidic protein (GFAP), and these proteins may be considered to be brain-specific biomarkers that can be used to assess brain damage caused by a ruptured aneurysm in the acute and long-term phases of treatment.

Outcome prediction using clinical scores recognized in neurological critical care, such as the Glasgow Coma Scale score, WFNS, and Hunt and Hess grade, has been useful in patients with SAH. However, the clinical evaluation is of limited value to ICU physicians, when there is prolonged sedation of a patient that is often required to treat elevated intracranial pressure or in patients on mechanical ventilation. The scientific evidence to date suggests that SAH-associated brain injury is a complex, multifactorial process, where early brain damage affects secondary damage and the end result of treatment. Thus, independent mechanisms from vasoconstriction, such as early brain damage, spreading depolarization, oxidative stress, inflammation, and blood–brain barrier disruption, can have a much greater effect on delayed cerebral ischemia and outcome than the mere presence of cerebral vasospasm [6, 7]. Defining a panel of brain-specific biomarkers that would reflect the degree of brain damage could be useful in planning and determining therapeutic directions at the ICU. In the early phase after aSAH, reliable laboratory tools for outcome prediction could be particularly helpful and could facilitate the management of the patient. In very few studies, the patient outcome was evaluated in the acute phase of aSAH treatment in relation to the patient’s clinical condition at discharge from the ICU; most studies evaluated the predictive value of brain biomarkers in relation to the long-term outcome after SAH. The primary goal of this paper was to predict outcome within the very early phase after SAH. Analysis was done to identify specific biomarkers that are predictors of a bad outcome at ICU discharge. Early changes in brain-specific biomarker levels were assessed in patients after aSAH, in the acute phase of treatment, in relation to clinical findings, severity scores, and ICU outcome.

## Materials and Methods

### Study Population

This observational, prospective study included patients with aSAH admitted to the Intensive Care Unit (ICU) at the University Hospital in Wroclaw between July 2014 and January 2017.

1. Inclusion criteria: age ≥ 18 years, ICU admission within 48 h after clinical diagnosis of aSAH. An aneurysm in cerebral arteries as the cause of bleeding was confirmed using computer tomography angiography (CTA), magnetic resonance imaging (MRI) or conventional angiography,
2. Exclusion criteria: previously diagnosed neurological disease.

Patients with previously diagnosed aneurysms, with a history of stroke, epilepsy, neurodegenerative diseases such as Parkinson’s disease, dementia, cerebrovascular diseases, motor neuron diseases, traumatic brain injury, schizophrenia, or depression were excluded. Exclusion criteria were based on previously published data, indicating that levels of some brain-specific biomarkers may be elevated in these diseases [8–12].

### Outcome Assessment, Study Groups

The neurological condition of the patients was graded according to the 5-grade Glasgow Outcome Scale (GOS). The GOS is the most commonly used outcome measurement after an acute brain injury. To calculate the GOS, the best source of available information should be used. Therefore, for this study, the parameters of patient conditions at discharge from the ICU were used to calculate the GOS, and the GOS results were dichotomized into two categories: good or bad outcome.

**Study Group 1** consisted of patients with a good outcome (GOS IV–V).

**Study Group 2** consisted of patients with a bad outcome (GOS I–III) (Table 1).

**Table 1:** Classifying study groups based on the Glasgow Outcome Scale (GOS)

Study groups	GOS rating	Definition
Group 1	5: Good recovery	Resumption of normal life despite minor deficits
	4: Moderate disability	Disabled but independent. Can work in sheltered setting
Group 2	3: Severe disability	Conscious but disabled. Dependent for daily support
	2: Persistent vegetative	Minimal responsiveness
	1: Death	Nonsurvival

### Clinical Evaluation and Management

Patients were given treatment according to a standardized management protocol [13], and study procedures were previously described in detail [14]. Briefly, all patients diagnosed with a subarachnoid hemorrhage caused by a ruptured **c**erebral aneurysm were admitted to the ICU. Neurosurgical intervention was undertaken within the first 24 h after aSAH. The decision on the type of procedure (surgical clipping or endovascular coiling,) as well as the time of the procedure was made by a team of neurosurgeons and interventional neuroradiologists based on medical indications, such as the results of the complete blood count, size and location of the aneurysm, and the neurological status of the patient. In patients assessed as a IV or V on the WFNS scale, for early management of hydrocephalus and elevated intracranial pressure, external continuous ventricular drainage (EVD) with of the cerebrospinal fluid was first implanted. Then, after stabilization of the general and neurological condition, endovascular coiling was performed. In patients with uncontrolled elevated intracranial pressure (ICP), due to an intracerebral hematoma or edema, open surgical clipping was done. The decision to place the sensor for measuring intracranial pressure, EVD or decompressive craniectomy was made individually for each patient. After securing the aneurysm, the EVD was removed as quickly as clinically feasible. Most often, intermittent drainage of cerebrospinal fluid was used, with an early attempt to clamp. At the ICU, all treatment algorithms included euvolemia, analgesia, sedation, and inotropic support, when indicated. Nimodipine was administered to all patients with aSAH to improve neurological outcome. Depending on the indications, patients were either mechanically ventilated or they remained on passive oxygen therapy. A control CT scan was performed within 24 h after excision or collapse of the aneurysm. Neurological examinations were performed by an ICU physician daily to detect neurological impairment (movement disorders, mental disorders, aphasia). Demographic data, medical history, and baseline clinical parameters were obtained shortly after admission. At the ICU, the patient’s clinical status was assessed with the APACHE II score (Acute Physiology and Chronic Health Evaluation II). Neurological status was assessed with the GCS (Glasgow Coma Scale). To classify the severity of aSAH based on the patient’s clinical condition, the Hunt–Hess scale was used, and the extent of the hemorrhage on the CT was graded with the Fisher scale [15]. Additionally, the patient’s follow-up was done with a Glasgow Outcome Scale (GOS) at hospital discharge and with a Glasgow Outcome Scale Extended (GOSE) after 6 months.

### Blood Sample Collection and Biomarker Detection

For each patient, blood samples were collected at the time of admission (day 1), and on day 2, 3, 4, 5, and 6 after aSAH. A blood sample was drawn from an intravenous catheter to a tube (2.7 mL). Each blood sample was centrifuged after 30 min (10,000 rpm for 15 min), and the supernatant was aliquoted and stored at − 70 °C until assayed. A solid phase enzyme linked-immuno-sorbent assay (ELISA) was used to measure serum levels of S100B (Cloud-Clone Corp., Katy, TX, USA), GFAP (Elabscience, Houston, Texas, USA), MAPT (Cusabio TECHNOLOGY LLC, Houston, TX, USA), and NSE (R&D Systems, Minneapolis, MN, USA). Concentrations of mediators were measured in duplicate with appropriate controls, according to the manufacturer’s instructions using an ELx800 absorbance microplate reader (BioTek, Winooski, VT, USA).

### Statistical Analysis

All analysis was performed with Statistica 13 software (StatSoft, Inc. Tulsa, USA). The distribution was not normal based on the Shapiro–Wilk test. Therefore, statistical analysis was performed using nonparametric tests. Comparisons of biomarker levels within a single group among different time points (day 1, 2, 3, 4, 5, and 6) were performed using the Friedman analysis of variance (ANOVA) and Kendall’s coefficient of concordance test. Categorical variables were analyzed using the Chi-square test. The Mann–Whitney U test was used for comparison of continuous variables between study groups at each time point. The Spearman rank test was used for correlations. A comparison of the predictive accuracy of the biomarkers measured on admission to the ICU was made using receiver operating characteristics curve (ROC) analysis, by calculating the area under the curve (AUC), including 95% confidence intervals (CI), to determine sensitivity and specificity. Multivariate logistic regression analysis was performed to evaluate the association between baseline S100B, NSE, MAPT, and GFAP and covariates age, gender, GCS, APACHEII, WFNS scale, Hunt and Hess scale, Fisher scale) and ICU outcome; the results were reported as odds ratio (OD) and 95% confidence intervals (CI). The first prepared model included all biomarkers and all selected covariates (age, gender, GCS, APACHEII, WFNS scale, Hunt and Hess scale, Fisher scale).The collinearity of the variables was tested. Four of the covariates (GCS, WFNS scale, Hunt and Hess scale, Fisher scale) were collinear, so those features were excluded from the analysis. The choice of the best model was proposed based on the Akaike information criterion and the backward selection of the model. From the four biomarkers (S100B, NSE, MAPT, and GFAP) and the three covariates (age, gender, APACHEII), the procedure of minimizing Akaike criterion chose the model with two biomarkers (S100B, NSE) and two covariates (APACHEII, gender). The statistical analysis was conducted using R 3.6.01: R Core Team (2013). Continuous variables were reported as mean values ± standard error and minimum–maximum. All the tests were conducted with a 5% significance level.

## Results

Out of the 60 patients with aSAH who met the inclusion criteria, 5 were excluded due to an incomplete acquisition of samples. The analysis was performed on 55 patients (Group 1, *N* = 24 and Group 2, *N* = 31). The mean admission GCS was 11.6 (range 4–15), a WFNS grade of I–III was recorded in 36 patients (65%) and a WFNS grade of IV–V in 19 patients (35%). The results of the general clinical assessment scores WFNS, GCS, Hunt–Hess, and the Fisher scale were significantly better in Group 1 than in Group 2. The APACHE II score, used for the classification of disease severity in ICU patients, was significantly lower in Group 1, indicating a better clinical status on admission to the ICU (Group 1: 9.6 ± 0.8 pts, Group 2: 17.1 ± 1.2 pts., *p *< 0.001). A summary of patient baseline characteristics is given in Table 2.

**Table 2:** Characteristics of the study population on admission to the ICU

Parameter	All (*N* = 55)	Group 1 (*N* = 24)	Group 2 (*N* = 31)	*p*
Age (years)	58.8 ± 1.9 (25–83)	52.9 ± 2.0 (25–82)	63.3 ± 2.4 (30–83)	0.005
Gender (F/M)	36/19	17/7	19/12	0.460
APACHE II	13.8 ± 0.9 (2–29)	9.6 ± 0.8 (2–21)	17.1 ± 1.2 (4–29)	< 0.001
WFNS scale [*n* (%)]				0.020
I–III	36 (65)	20 (83)	16 (52)	
IV–V	19 (35)	4 (17)	15 (48)	
Initial CT Fisher scale (subarachnoid blood) [*n* (%)]				0.002
Grade I (none)	0	0	0	
Grade II (diffuse only)	12 (22)	10 (42)	2 (6)	
Grade III (clot or thick layer)	15 (27)	7 (29)	8 (26)	
Grade IV (diffuse or none, with cerebral or ventricular blood)	28 (51)	7 (29)	21 (68)	
GCS	11.6 ± 0.54 (4–15)	13.6 ± 0.4 (5–15)	10.0 ± 0.8 (4–15)	< 0.001
Hunt and Hess scale, (the severity of subarachnoid hemorrhage) [*n* (%)]				0.011
Grade I	10 (18)	8 (33)	2 (6)	
Grade II	8 (15)	4 (17)	4 (13)	
Grade III	15 (27)	8 (33)	7 (23)	
Grade IV	10 (18)	3 (15)	7 (23)	
Grade V	12 (22)	1 (4)	11 (35)	
Treatment [*n* (%)]				0.227
Neurosurgical clipping	23 (42)	9 (38)	14 (45)	
Endovascular embolization	27 (49)	14 (58)	13 (42)	
Conservative	5 (9)	1 (4)	4 (13)	

Data are presented as mean± standard error (min–max), unless other stated; CT, computed tomography; GCS, Glasgow Coma Score; LOS, length of stay; WFNS, the World Federation of Neurological Surgeons; *p* value represents a comparison between groups

### Aneurysm Treatment

All patients had an aneurysm in a cerebral artery which was confirmed as the cause of bleeding using computer tomography angiography (CTA), magnetic resonance imaging (MRI) or conventional angiography. Twenty-three patients (42%) underwent neurosurgical clipping of the aneurysm, 27 (49%) underwent endovascular embolization, and the other patients were treated conservatively (*N* = 5, 9%) (Table 2). Patients treated conservatively (H–H score 5, Fisher score 4) had only external ventricular drain (EVD) in the acute phase of treatment as decided by the attending neurosurgeons. Lack of improvement in the neurological condition was the reason for disqualification of these patients from further neurosurgical interventions. There was no statistically significant difference in the distribution of the treatment methods between Group 1 and Group 2 (*p *= 0.227). Patients, who underwent either embolization or clipping, did not differ significantly in age, gender, and the results of the clinical scores on admission to the ICU (APACHE II *p *= 0.952, WFNS *p*-0.912, GCS *p *= 0.629, Hunt and Hess *p *= 0.503, and Fisher scale *p *= 0.401). S-100B, NSE, GFAP, and MAPT levels recorded on days 1, 2, 3, 4, 5, and 6 were not significantly different in patients whose aneurysms were coiled and clipped. The Glasgow outcome score at ICU discharge was not different for patients who underwent clipping or embolization (respectively, 2.95 ± 0.239 and 3.18 ± 0.238, *p *= 0.451). Vasospasm occurred in 15 patients (62%) in Group 1 and in 18 patients (58%) in Group 2 (*p *= 0.739). S-100B, NSE, GFAP, and MAPT levels were not significantly different in patients with and without vasospasm.

### Biomarker Kinetics in the Acute Treatment Phase

**Fig. 1:**
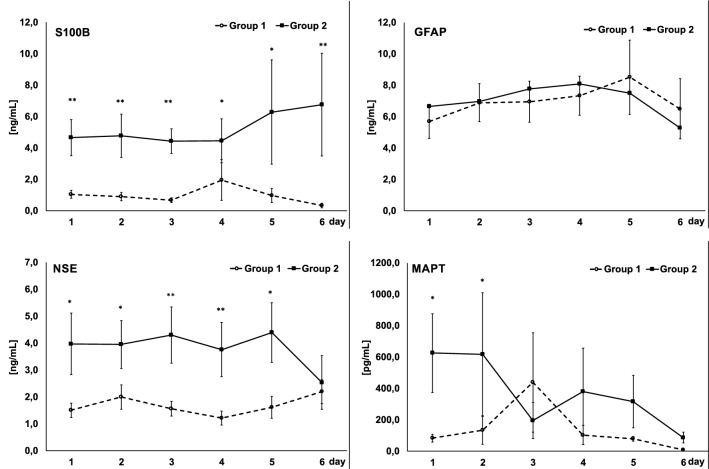
Temporal course of S100B, glial fibrillary acidic protein (GFAP), neuron-specific enolase (NSE), and microtubule-associated protein tau (MAPT) in patients with a good outcome (Group 1) and in patients with a bad outcome (Group 2), based on the grade in the Glasgow Outcome Scale at discharge from the ICU. (*p < 0.05; **p < 0.001)

S100B, MAPT, and NSE levels were correlated with the neurological outcome of the patients evaluated with the GOS at discharge from the ICU. At baseline, there was a significant increase in the level of the biomarkers in the group of patients with a bad outcome (Group 2) but not in Group 1 (S100B: 1,0 ± 0.3 vs. 4.7 ± 0.1.1 ng/mL; MAPT: 83.2 ± 25.1 vs. 625,0 ± 250.7 pg/mL; NSE: 1.5 ± 0.3 vs. 4.0 ± 1.2 ng/mL in Group 1 and 2, respectively). In Group 2, S100B and NSE levels remained significantly elevated during the whole study period (S100B: 4.8 ± 1.4, 4.4 ± 0.8, 4.5 ± 1.3, 6.6 ± 3.3, 6.8 ± 3.2 ng/mL; NSE: 4.1 ± 0.9, 4.3 ± 1.1, 3.8 ± 1.0, 4.4 ± 1.1, 2.5 ± 1.1 ng/mL on day 2, 3, 4, 5, and 6, respectively) and stayed low in Group 1 (S100B: 0.9 ± 0.3, 0.7 ± 0.1, 2.0 ± 1.3, 1.0 ± 0.4, 0.3 ± 0.2 ng/mL, NSE: 2.0 ± 0.5, 1.6 ± 0.4, 1.2 ± 0.3, 16.6 ± 0.4, 2.2 ± 0.4 ng/mL on day 2, 3, 4, 5, and 6, respectively). The MAPT level remained elevated only until the second day of observation in Group 2 (617.4 ± 391.6, 195.0 ± 115.6, 379.9 ± 277, 315.5 ± 167.0, 86.9 ± 34.8 pg/mL on day 2, 3, 4, 5, and 6, respectively) and stayed low in Group 1 (132.7 ± 88.1, 438.7 ± 316.9, 102.9 ± 62.2, 79.7 ± 14.7, 8.1 ± 8.0 pg/mL on day 2, 3, 4, 5, and 6, respectively). The difference between groups in S100B and NSE was significant during the whole study period, and the difference in the MAPT level between groups was significant on day 1 and 2. An aSAH increased the blood concentration of GFAP significantly from day 1 and remained high till the end of the observation period (Group 1: 5.7 ± 1.5, 6.9 ± 1.6, 6.9 ± 1.5, 7.3 ± 1.5, 8.5 ± 1.7, 6.5 ± 3.3 ng/mL; Group 2: 6.6 ± 1.1, 7.0 ± 1.2, 7.8 ± 1.3, 8.1 ± 1.2, 7.5 ± 2.4, 5.3 ± 1.9 ng/mL on day 1, 2, 3, 4, 5, and 6, respectively); however, no significant differences were observed between the groups studied. The temporal course in biomarkers in Groups 1 and 2 is presented in Fig. 1. No significant correlation was found between a patient’s age or gender and the levels of GFAP, MAPT, S100B or NSE.

### Prediction of Clinical Outcome After aSAH

Based on the value of the GOS recorded upon discharge from the ICU, 44% of the study population had a good outcome (Group 1, GOS IV–V) and 56% had a bad outcome (Group 2, GOS I–III). The majority of patients within Group 1 were graded with a GOS of IV (79%), and in Group 2, the majority were graded with a GOS of III (61%). Table 3 displays a comparison between patients with a good versus bad clinical status based on the GOS recorded at ICU discharge. There were no patients with a grade 2 on the GOS.

**Table 3:** A comparison of patients with a good (Group 1) versus poor (Group 2) status based on the value of the GOS recorded at discharge from the ICU

Parameter	Group 1 (*N* = 24)	Group 2 (*N* = 31)	*p*
GOS at ICU discharge [*n* (%)]			NA
Grade 1	–	12 (39)	
Grade 3	–	19 (61)	
Grade 4	19 (79)	–	
Grade 5	5 (21)	–	
ICU LOS (day)	10.4 ± 1.7 (2–44)	17.9 ± 2.5 (3–62)	0.029
Hospital LOS (day)	25.3 ± 6.5 (9–145)	52.3 ± 7.1 (9–120)	< 0.001
ICU survival [*n* (%)]	24 (100)	19 (61)	< 0.001
Hospital survival [*n* (%)]	24(100)	18 (58)	0.001
Patient’s status, discharge from hospital [*n* (%)]			< 0.05
Dead	0	13 (42)	
Home	20 (83)	13 (42)	
Rehabilitation ward	1 (4)	2 (6)	
Another hospital	3 (13)	3 (10)	
Follow-up:			
GOS at hospital discharge [*n* (%)]			< 0.001
Grade 1	–	13 (42)	
Grade 2	–	1 (3)	
Grade 3	–	7 (22)	
Grade 4	6 (25)	9 (29)	
Grade 5	18 (75)	1(3)	
GOSE at 6-month follow-up [*n* (%)]			< 0.001
Grade 1	1 (4)	15 (48)	
Grade 4	–	2 (6)	
Grade 5	1 (4)	7 (23)	
Grade 6	2 (8)	2 (6)	
Grade 7	12 (50)	4(13)	
Grade 8	8 (33)	1 (3)	

Data are presented as mean± standard error (min–max), unless other stated; GOS, Glasgow Outcome Scale; GOSE, Glasgow Outcome Scale Extended; LOS, length of stay; *p* value represents a comparison between groups

Based on the value of the GOS recorded at hospital discharge, the majority of patients within Group 1 (good ICU outcome) were graded with a GOS of V (75%) indicating a good hospital outcome; in Group 2 (bad ICU outcome), 42% of patients died (GOS of I), and among the survivors, the majority were graded with a GOS of III and IV (22% and 29%, respectively) (Table 3). At 6-month follow-up, based on the value of the Glasgow Outcome Scale Extended (GOSE), the majority of patients within Group 1 were graded with a GOSE of VII (50%) and VIII (33%) indicating improvement in the clinical condition and only one patient died (GOSE of I). In Group 2, there were two additional deaths (15 deaths in total, 48%), and the majority of survivors were graded with a GOSE of IV (6%) or V (23%) indicating severe or moderate disability, respectively (Table 3).

**Fig. 2:**
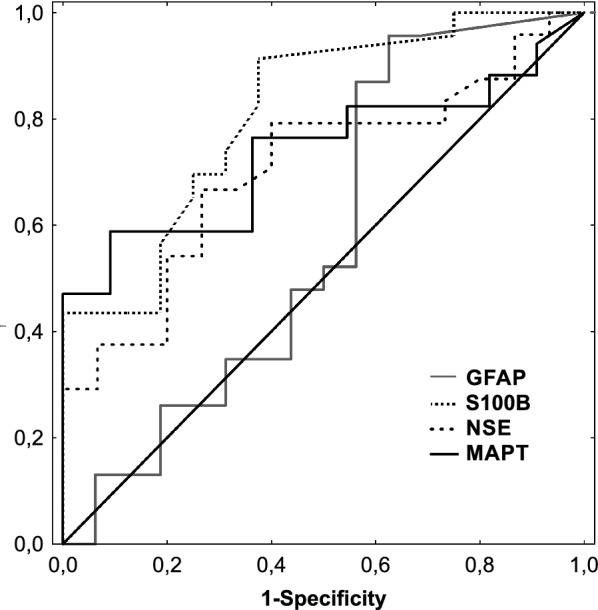
Receiver operating characteristic curves for outcome prognosis of the baseline S100B (AUC 0.813), neuron-specific enolase (NSE; AUC 0.706), microtubule-associated protein tau (MAPT; AUC 0.735), and glial fibrillary acidic protein (GFAP; AUC 0.575) levels in the serum of patients after an aneurysmal subarachnoid hemorrhage

The receiver operating characteristic curves for outcome prognosis of the baseline S100B, NSE, MAPT, and GFAP in the serum of patients after an aneurysmal subarachnoid hemorrhage are shown in Fig. 2. In the ROC curve analysis, S100B (AUC 0.813; 95% CI 0.677–0.948, *p *< 0.001) showed the highest ability to predict bad ICU outcome among the single biomarkers measured on admission. The optimal threshold value for the baseline S100B was 0.625 ng/mL, with sensitivity of 0.913 (95% CI 0.833–1.067) and specificity of 0.625 (95% CI 0.651–1.249). The baseline NSE also had significant predictive value (AUC 0.706; 95% CI 0.541–0.871, *p *= 0.015); however, for the optimal threshold value of 1.613 ng/mL, the sensitivity (0.667; 95% CI 0.719–1.180) of the marker was no longer as good as for S100B, and specificity was 0.733 (95% CI 0.689–1.211). The baseline MAPT had significant predictive value (AUC 0.735; 95% CI 0.550–0.892, *p *= 0.012); for the optimal threshold value of 240.7 pg/mL, the sensitivity was low (58.8%; 95% CI 0.647–1.253), but the specificity of the marker was very good (0.909; 95% CI 0.830–1.070). The baseline GFAP prediction value was not significant (*p *= 0.46).

In addition, a multivariate logistic regression analysis was performed to create a model predicting bad outcome. The choice of the variables from the set of biomarkers (S100B, NSE, MAPT, and GFAP) and covariates (age, gender, GCS, APACHEII, WFNS scale, Hunt and Hess scale, Fisher scale) was determined by the minimizing of the Akaike information criterion and the backward selection of the model; collinear covariates were excluded from the analysis. Therefore, the only variables that were included in the final model were the initial S100B, NSE, gender, and APACHEII. The initial S 100B, APACHEII, and gender were significant predictors of bad outcome. The initial NSE had no statistical significance in the model (*p *= 0.088). Results of analysis are presented in Table 4.

**Table 4:** Results of a multivariate logistic regression analysis model predicting bad ICU outcome in patients after aSAH

	Odds ratio	95% confidence intervals	*p* value
S100B	2.229	1.275–5.168	0.023
NSE	1.354	1.062–2.020	0.088
APACHE II	1.303	1.119–1.601	0.002
Gender	0.089	0.008–0.575	0.022

### Correlations of Brain Biomarkers with GOS

At ICU discharge, a strong negative correlation between the GOS and the level of S100B was recorded on days 1–6, between the GOS and the level of NSE on days 1–5 (Table 5), and between the GOS and the level of MAPT on day 1; there was no correlation between GOS and GFAP. There was no correlation between any of the biomarkers measured on day 1, and the severity scores recorded on admission to the ICU (APACHE II, GCS, H–H, Fisher, and WFNS).

**Table 5:** *The Spearman’s* rank *correlation coefficient* between neuronal biomarkers and the Glasgow Outcome Scale

Day	S100B and GOS	NSE and GOS	MAPT and GOS	GFAP and GOS
1	− 0.6**	− 0.4*	− 0.4*	n.s.
2	− 0.5**	− 0.4*	n.s.	n.s.
3	− 0.7**	− 0.6**	n.s.	n.s.
4	− 0.4*	− 0.5**	n.s.	n.s.
5	− 0.6*	− 0.5*	n.s.	n.s.
6	− 0.8*	n.s.	n.s.	n.s.

*p < 0.05; **p < 0.001, n.s. statistically non-significant

## Discussion

In this report, we describe a panel of proteins abundant in the cells of the central nervous system that increase in the blood following an aSAH, and they may be early predictors of the neurological outcome in the acute treatment phase. In this study, we limited the question of outcome prognostication to the acute phase of aSAH treatment, i.e., at discharge from the ICU, and the biomarker concentrations were evaluated for six consecutive days after aSAH. Evaluating aSAH patients using clinical scores becomes more difficult with ICU patients receiving sedatives and analgesics. Biomarkers assessments might be an independent, additional tool to support the clinical evaluation at the ICU. Analysis was performed for the proteins of neurofilament, neuron-specific enolase (NSE) and microtubule-associated protein tau (MAPT), and for the proteins of glial cells, S100B and glial fibrillary acidic protein (GFAP). Outcomes were assessed at discharge from the ICU and analyzed based on the grade in the Glasgow Outcome Scale. Patients were classified into two groups: patients with a good outcome (GOS IV–V) and patients with a bad outcome (GOS I–III). The main findings are: (1) brain biomarkers S100B, NSE, GFAP, and MAPT increase markedly following an aSAH, (2) the baseline S100B shows the highest ability to predict bad ICU outcome after aSAH, and (3) in acute phase of aSAH treatment, S100B and NSE correlates with neurological outcome.

For practical reasons, all biomarker measurements were performed in serum samples. A high level of neuronal biomarkers in the blood of patients with an aSAH is a consequence of the damage to the neurons and to the blood–brain barrier, in which biomarkers are allowed to pass from the cerebrospinal fluid into the bloodstream. The ability to measure the level of brain damage markers in serum instead of in the cerebrospinal fluid seems more useful and universal, because patient blood samples can be collected regardless of elevated intracranial pressure. Moreover, it has been found that the duration of external ventricular drainage (EVD) and CSF sampling frequency were significant risk factors for EVD-related infections, i.e., meningitis or ventriculitis (Hoefnagel et al. [16].

S100B, NSE, GFAP, and MAPT have previously been used as markers of neuronal damage, such as in stroke [17, 18], traumatic brain injury [19, 20], and aneurysmal SAH [21, 22]. Though a substantial number of studies have been performed to examine serum and CSF levels in patients with a range of neurological disorders, findings have been inconsistent regarding the changes in concentration of brain-specific biomarkers after an aneurysmal type SAH. Several clinical investigations have shown that the concentrations of S100B, NSE, GFAP, and MAPT are elevated in the serum and CSF samples of patients with an aSAH; however, large variations in the concentrations of the markers were reported. In an early study by Nylén et al. [23], increased s-GFAP levels were seen in a majority of aSAH patients, but the correlation with a WFNS was weak on admission (correlation coefficient < 0.4). Vos et al. [24] demonstrated increased glial (S100b and GFAP) but not neuronal (NSE) protein levels in peripheral blood at hospital admission. Additionally, high S100b and GFAP serum concentrations found after SAH were associated with the clinical severity of the initial injury, as measured by the WFNS scale. In another study, an early temporal profile of the S100B concentration after an aSAH was characterized by peak initial values followed by a decrease during the ensuing days post-injury [25]. Moreover, a threshold of initial S100B levels of > 0.7 μg/dl in serum was associated with 100% mortality. Our results also show large variation in concentrations of all the tested markers, from negligible to very high. However, certain kinetic patterns can be identified. Changes in S100B and NSE can be characterized with a similar pattern: S100B and NSE increased significantly at baseline and remained elevated throughout the study period in the group of patients with a bad outcome, while it stayed low in patients with a good outcome. The MAPT level was significantly elevated during the first 2 days of observation in the group of patients with a bad outcome, while it remained low in patients with a good outcome; the fifth and sixth day of observation had a similar pattern: the mean MAPT level decreased in both study groups. The mean GFAP level was elevated throughout the study period, and this pattern was seen in both groups, without significant differences in the kinetics. It should be noted that the initial hemorrhage in aSAH patients can be accompanied by a number of other factors, including intracerebral hematomas, re-bleedings, secondary ischemic events, and complications after surgery. All these factors may contribute to changes in the brain-specific biomarkers measured in the serum at different points in time. According to the multivariate logistic regression analysis, the best model predicting bad ICU outcome after SAH included initial S100B, NSE, APACHEII score and gender. An elevated S100B, and APACHE II score, and male gender indicated a significantly higher risk of bad ICU outcome, resulting in severe disability, persistent vegetative state or death (GOS rating 1–3).

Findings of other authors regarding the prognostic potential of S100B, NSE, GFAP, and MAPT after an aSAH have been inconsistent. Our results indicate that S100B and NSE were strongly associated with neurological outcome measured with the GOS at discharge from the ICU. In the group of patients with a bad outcome, S100B and NSE levels increased significantly at baseline and remained elevated throughout the study period. The strong relationship of serum S100B, NSE, and MAPT levels with patient status at ICU discharge (AUC = 0.813, AUC = 0.706, AUC = 0.735, respectively) indicates that these two proteins may be potential predictors of a bad outcome in the clinical setting. We found that baseline S100B with a cutoff value of 0.625 ng/mL provided very good sensitivity of 91.3% and the NSE with a cutoff value of 1.613 ng/mL provided a sensitivity of 66.7% in predicting bad ICU outcome. It was notable that the baseline MAPT provided a specificity of 90.9% in predicting a good ICU outcome.

Our findings are in line with some previously published results. However, in very few studies was patient outcome evaluated in the acute phase of aSAH treatment, i.e., at discharge from the ICU; most studies evaluated the predictive value of brain biomarkers in relation to the long-term outcome after SAH. Based on earlier data on the utility of measuring biomarkers with aSAH, there is some controversy regarding the threshold value for the prediction of a bad outcome. Moritz et al. [26] found that mean and peak values of S100B (cutoff 0.17 and 0.23 µg/L, respectively) provided the ability to distinguish between patients with good and bad outcome at ICU discharge, while NSE did not provide predictive value.

In a study of fifty-one SAH patients, an S100B that was higher than 1 µg/L within the first 3 days after SAH was predictive of an unfavorable outcome at 6-month follow-up, and the NSE concentration was not related to the outcome [27]. Sanchez-Peña et al. [28] observed that an elevated level of S100B over the first 15 days after a subarachnoid aneurysmal hemorrhage was associated with a bad outcome after SAH at 12-month follow-up and the best cutoff for the mean 15-day S100B value was 0.23 µg/L (specificity 90%, sensitivity 91%), which was much lower than in the study by Oertel and in our study. Abboud et al. [29] found that S100B and NSE levels measured daily for the first 3 days after a hemorrhage accurately predicted the neurological outcome in poor-grade aSAH patients at 6-month follow-up. The best cutoff for the mean 3-day S100B value was 1.172 µg/L with a specificity of 75%, and for the mean 3-day NSE value 14.6 µg/L, with a specificity of 71.4%.

Quite opposite results have recently been published. Kiiski et al. [30] found that S100B and NSE measured during the first 24 h were not associated with neurological outcome evaluated at 6 months after an aSAH. Similar results were published by Olivecrona et al. [31]; based on the biomarker concentrations determined twice daily for five consecutive days, there was no significant clinical value of S100B and NSE as predictors of clinical outcome at 3 and 12-month follow-up.

As shown above, the time at which the determination of the biomarker concentrations would have the highest prognostic value and be of the greatest importance in identifying patients at risk of poor results remains a matter of dispute. Moreover, different values were used for the analyses: the value from the first day only, the mean of all measurements, the peak value. Different follow-up points were taken into consideration: short-term, such as ICU discharge, hospital discharge, 3-month follow-up, and long-term, such as 6- or 12-month follow-up.

The significant differences in the published results and the importance of biomarkers in predicting long-term clinical outcome after an aSAH may be also due to differences in the management of patients after the completion of ICU treatment. Rehabilitation content is a challenge, requires interdisciplinary cooperation, and may vary depending on the patient’s clinical status. In addition, rehabilitation programs specializing in neurologic disorders after an aSAH may differ from country to country and may not be equally available to patients.

The limitation of this study is the relatively small size of the group, and a larger population of patients should be included to increase the statistical power of the findings. Like most previous studies, our study is based on the experiences from one clinical center; therefore, it may not be possible to generalize to a larger population.

## Conclusions

Detecting the initial S100B, NSE, and MAPT in blood samples may prove to be a valuable diagnostic and prognostic tool in the very early phase of aSAH treatment. Tools for early outcome prediction in individual patients with SAH are needed, especially in the population of ICU patients receiving sedatives and analgesics, to more accurately assess clinical status, to direct care, and provide families with the most accurate information. Our findings provide further evidence that brain biomarkers such as S100B, NSE, GFAP, and MAPT increase markedly in patients following an aSAH. There is a direct relationship between the neurological outcome in the acute treatment phase and the levels of S100B, NSE, and MAPT. Measuring biomarkers should be considered as a potential additional tool, supporting but not replacing the results of clinical scales such as the APACHEII, WFNS, GCS, Hunt–Hess scale, the Fisher scale, and GOS.

## Abbreviations

aSAH: Aneurysmal subarachnoid hemorrhage
GFAP: Glial fibrillary acidic protein
MAPT: Microtubule-associated protein tau
NSE: Neuron-specific enolase
